# First report of a Tailless Whip Scorpion in Cyprus: the case of *Saraxioanniticus* (Kritscher, 1959) (Amblypygi, Charontidae)

**DOI:** 10.3897/BDJ.13.e157478

**Published:** 2025-07-10

**Authors:** Michael Hadjiconstantis, Matthew Stephen Smith, Christos Zoumides

**Affiliations:** 1 Association for the Protection of Natural Heritage and Biodiversity of Cyprus, Nicosia, Cyprus Association for the Protection of Natural Heritage and Biodiversity of Cyprus Nicosia Cyprus; 2 Department of Forests, Ministry of Agriculture, Rural Development and Environment, Nicosia, Cyprus Department of Forests, Ministry of Agriculture, Rural Development and Environment Nicosia Cyprus; 3 Cyprus bird-watching tours, Paphos, Cyprus Cyprus bird-watching tours Paphos Cyprus; 4 Energy, Environment and Water Research Centre (EEWRC), The Cyprus Institute, Nicosia, Cyprus Energy, Environment and Water Research Centre (EEWRC), The Cyprus Institute Nicosia Cyprus

**Keywords:** Amblypygi, *Saraxioanniticus*, *Charinusioanniticus*, citizen science, Tailless Whip Scorpions, Whip spiders

## Abstract

**Background:**

Amblypygi, commonly known as tailless whip scorpions or whip spiders, is an order of Arachnida noted for its cryptic habits and predominantly tropical and subtropical distribution. Although the group was first mentioned on the island of Cyprus in 1990, no specific taxonomic information was provided at the time.

**New information:**

Through a combination of literature review, targeted field surveys and citizen-science contributions, we confirm the presence of *Saraxioanniticus* (Kritscher, 1959) in Cyprus, representing the first documented record of this species on the island. Following seven years of dedicated searches, a live specimen was collected in 2023 and identified, thereby establishing *S.ioanniticus* as part of the island’s rich fauna. This finding extends the known range of *S.ioanniticus* within the eastern Mediterranean and underscores the importance of integrating biodiversity research with public engagement to detect elusive or under-reported taxa. Future studies should investigate the species’ local distribution, ecological requirements and potential conservation concerns on Cyprus.

## Introduction

Amblypygi, commonly known as Whip spiders or Tailless Whip Scorpions, are a small, ancient order of Arachnida, characterised by their very thin and elongated first pair of legs and flattened bodies. They are nocturnal predators, primarily feeding on insects and small invertebrates (Weygoldt 2000). This order is distinguished by their specialised first pair of legs, which act as sensory organs rather than for locomotion, aiding in prey detection (Harvey 2003). Furthermore, amblypygids differ from spiders by lacking venom glands in the chelicerae, secondary copulatory organs on the male palps and spinnerets on the opisthosoma (Dunlop 2010). In addition, they differ from other ‘whipped’ orders of arachnids, like whip scorpions (Uropygi) and Palpigradi, by the absence of a flagellum at the end of the opisthosoma (Weygoldt 2000, Harvey 2003). Amblypygi have a relatively wide geographical distribution, with species found in tropical and subtropical regions across the globe, including parts of the Mediterranean, where they inhabit caves and humid microhabitats (Miranda et al. 2021).

The Mediterranean and western Palearctic regions are home to several species of Amblypygi, although their diversity in these areas is relatively low compared to tropical regions. One of the most notable species in the Mediterranean is *Saraxioanniticus* (Kritscher 1959), which was originally described as *Lindosiellaioannitica*, later classified as *Charinusioanniticus* and, more recently, placed in the genus Sarax (Miranda et al. 2021). This species was first described from the Greek island of Rhodes (Kritscher 1959, Harvey 2003) and it has since been recorded on other Greek islands, such as Symi and Kos and in continental Greece, Egypt, Jordan, Israel, Italy and Turkey (Miranda et al. 2021). *Saraxioanniticus* is typically found in humid caves and crevices, but has also shown a tendency to inhabit human-made environments, such as buildings and bathrooms, which suggests a degree of synanthropy (Agapakis and de Miranda 2019). *Saraxioanniticus* is also known to reproduce through parthenogenesis, a form of asexual reproduction where females produce offspring without fertilisation. This reproductive strategy is advantageous for the species, allowing populations to persist even in isolated or low-density environments, as seen in various Mediterranean and Middle Eastern populations (Weygoldt 2007, Blick and Seiter 2016). Another species, *Saraxisraelensis* (Miranda et al. 2016), was described from Israel, where it inhabits caves in the Galilee and Golan Heights, further expanding the known distribution of Amblypygi in the region (Miranda et al. 2021, Baker et al. 2022). According to the latest global phylogenetic research on Amblypygi, the families Charontidae and Charinidae - the latter of which previously included the genus Sarax - have been merged into a single family, namely Charontidae (de Miranda et al. 2024).

In the western Palearctic, *Phrynichusdeflersi* Simon, 1887 is the most prominent species, occurring primarily in the Arabian Peninsula, including Saudi Arabia, Yemen and Oman (Weygoldt 1999a). Like *S.ioanniticus*, this species prefers humid environments and is often found in caves and rock crevices (Cave et al. 2009). Two noteworthy troglomorphic species that add to the regional diversity of the genus *Sarax* are *S.omanensis* (Cave et al. 2009), described from the Al Hoota–Al Fallah cave system in northern Oman and *S.dofarensis* (Weygoldt et al. 2002) from deep limestone caves on the Dhofar karst plateau of southern Oman. Both species are confined to the aphotic zones of their respective cave systems and exemplify the genus’ adaptation to extreme subterranean humidity. Additionally, the phrynichid *Damondiadema* (Simon, 1876) has been verified from Yemen and represents the northern‐most occurrence of this predominantly Afrotropical genus (Weygoldt 1999b). Finally, *Musicodamonatlanteus* Fage, 1939 is endemic to the Maghreb, with records in Morocco and Algeria, particularly in mountainous regions like the Atlas Mountains, and it primarily inhabits caves and rocky habitats, demonstrating a unique adaptation to its environment (Fage 1939, Harvey 2003). Despite the relative scarcity of Amblypygi species in these regions, their adaptability to both natural and anthropogenic habitats highlights their ecological importance and evolutionary resilience.

The presence of Amblypygi on the island of Cyprus was first reported by Boris Sket in a study on Isopoda of Cyprus. In that publication, Sket (1990) noted a site at the monastery of Agios Neofytos (northeast of Paphos) with a limestone collector gallery that enriched a natural spring, where carcasses of Amblypygi were found at the bottom. However, no specimens were collected at the time and no subsequent literature on Amblypygi in Cyprus has been published. The objective of this study is to verify, for the first time, the presence of Amblypygi in Cyprus, thereby contributing a new arachnid family and species to the island’s checklist and broadening regional knowledge of this order in the eastern Mediterranean.

## Materials and methods

Between July 2017 and May 2025, targeted surveys were carried out using hand torches, in habitats with high relative humidity (caves, abandoned wells, sewer outlets, shaded garden walls) all over the island. On 2 July 2023, one live adult specimen was collected from a plastered garden wall at Pissouri Village, Limassol District (34°40'08.8"N 32°42'02.3"E). The specimen was kept for three days in a ventilated terrarium, with coconut fibre substrate, dry leaf litter and some rocks with cracks and was provided water, moisture and small mealworms and cockroaches as food.

Three days later and after natural death, the specimen was fixed in 90% alcohol and stored in a freezer (personal collection of Michael Hadjiconstantis). The specimen was then brought to The Cyprus Institute in Nicosia, Cyprus and was examined using a Hirox KH-8700 digital microscope with MXG-2500REZ lens (35–2500×) on a high-precision motorised stand; z-stacked images were rendered with Hirox software. The diagnostic characters were matched against *Saraxioanniticus*, following the taxonomic key in Miranda et al. (2021).

## Taxon treatments

### Sarax ioanniticus

(Kritscher, 1959)

59BE74E6-70A7-5445-8547-53D540EEA836

https://www.gbif.org/es/species/2181422

#### Materials

- **Type status:** Other material. **Occurrence:** occurrenceRemarks: specimen walking on a house wall; recordedBy: Dr. Christos Zoumides; individualCount: 1; sex: female; disposition: stored in 90% ethanol in M. Hadjiconstantis private collection; occurrenceID: B844670D-388A-53C9-A04B-3D6FEA395667; **Taxon:** taxonID: https://www.gbif.org/es/species/2181422; **Location:** higherGeographyID: Limassol district; country: Cyprus; municipality: Limassol district; locality: Pissouri village; verbatimCoordinates: 34°40'08.8"N 32°42'02.3"E; **Event:** verbatimEventDate: 2 July 2023

#### Taxon discussion

**Figure 1.**
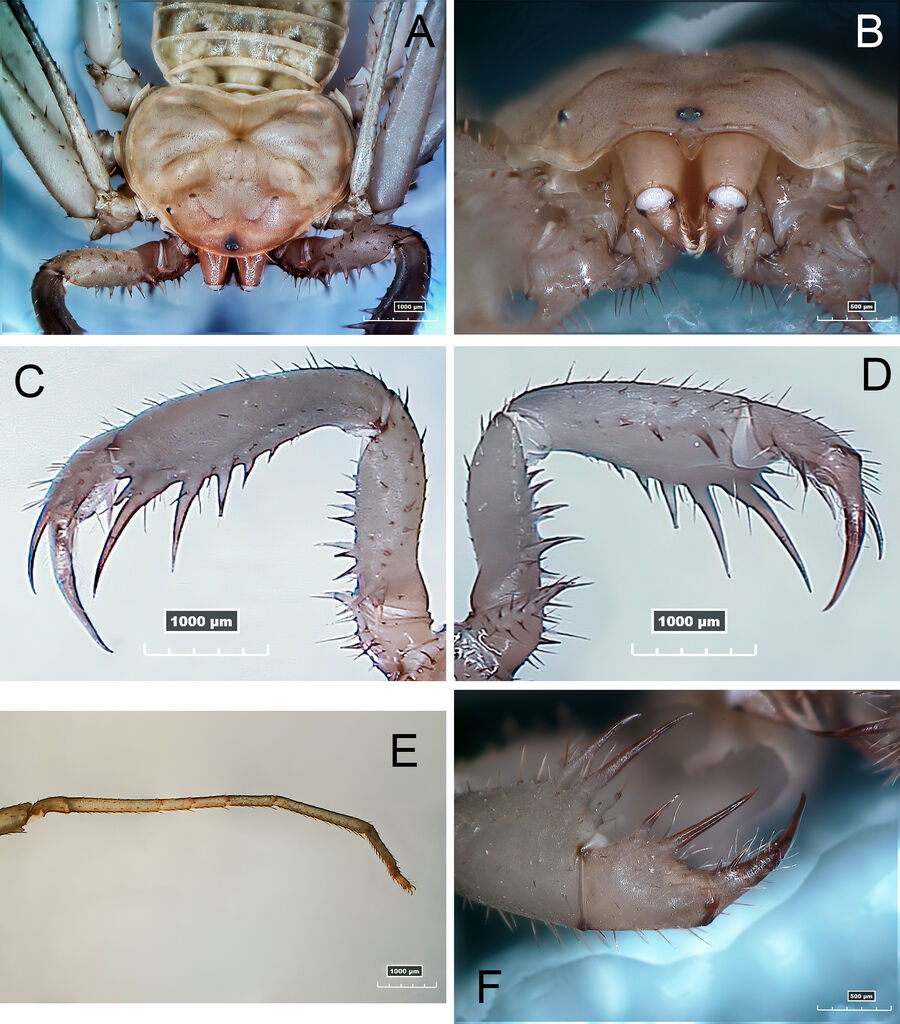
Morphological traits of *S.ioanniticus*: **A** carapace dorsal view; **B** carapace frontal view (frontal process); **C** pedipalp dorsal view; **D** pedipalp ventral view; **E** basitibia, distitibia and tarsus IV; **F** pedipalps’ tibia and tarsus frontal view.

**Figure 2.**
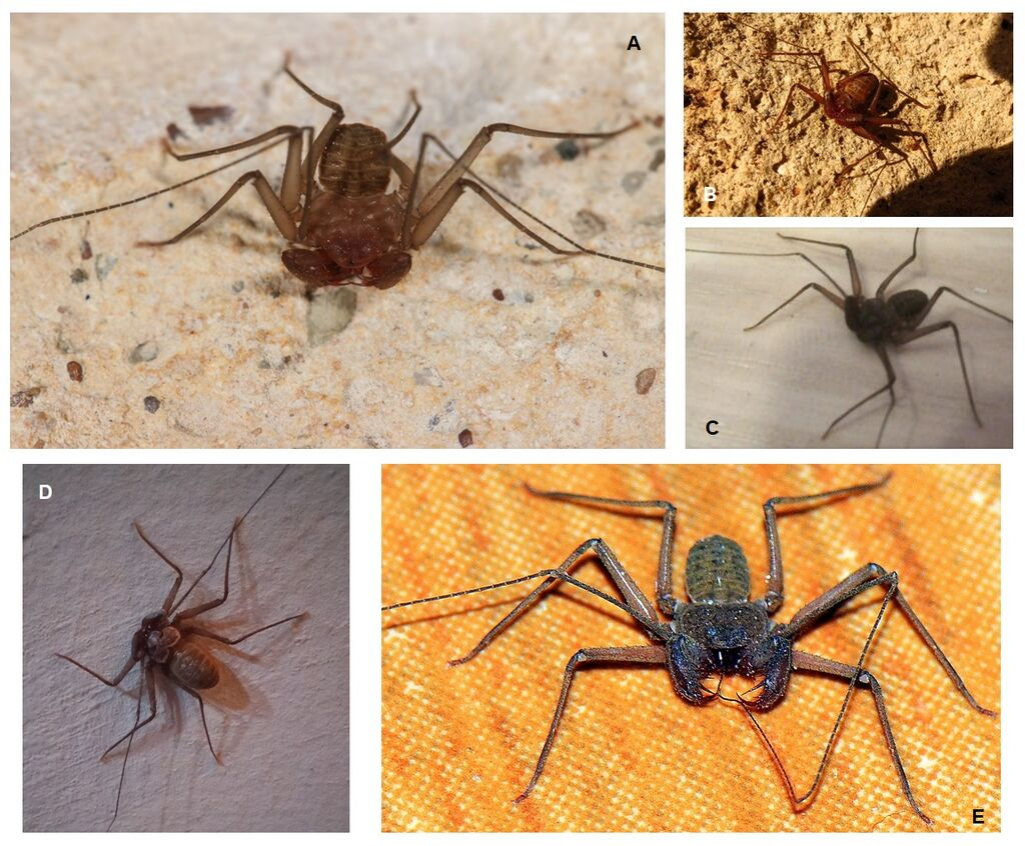
Habitus and online record photos of *S.ioanniticus* in Cyprus (photograph credits in parenthesis): **A** Habitus of the specimen from Pissouri (M. Hadjiconstantis); **B** Tsada (M. Smith); **C** Paphos city (M. Smith); **D** Lapithos (A. Proutheau); **E** Ortakioi (A. Gürses). The specimens B-E were not collected and the specific identification of the specimens is tentative.

The collected female specimen (Fig. 1, Fig. 2A) was identified asMiranda et al. 2021 *Saraxioanniticus*, based on the available taxonomic key in Miranda et al. (2021).

## Analysis

### Other Amblypygi records in Cyprus: citzen science and literature records

The following records are based on literature, photographs and video material posted on social media. No material was examined to confirm the species identification:

- Amblypygi sp., Holy Monastery of Saint Neophytos the Recluse, Tsada Village (34°50'48.0"N 32°26'44.7"E), Paphos District; Amblypygi carcasses on the bottom of a natural spring (Sket 1990).
- *Sarax* sp., Tsada Village (34°50'19.0"N 32°28'29.9"E), Paphos District; July 2011, 20+ specimens under the plates surrounding a pool, Photographer: Matthew Stephen Smith (Fig. 2B).
- *Sarax* sp., Kennedy Square (34°46'30.8"N 32°25'19.7"E), Paphos City; July 2012, video with a walking specimen, Videographer: Matthew Stephen Smith (Fig. 2C).
- *Sarax* sp., Lapithos Village (35°20'31.0"N 33°10'08.4"E), Kerynia District; 23 March 2021, Photo of a specimen on a house wall, Photographer: Aurore Proutheau (Fig. 2D).
- *Sarax* sp., Ortakioi Village (35°11'52.47"N 33°20'4.51"E), Nicosia District; 11 April 2025, Photo of a specimen on a house yard, Photographer: Ahmet Gürses (Fig. 2E).

**Figure 3.**
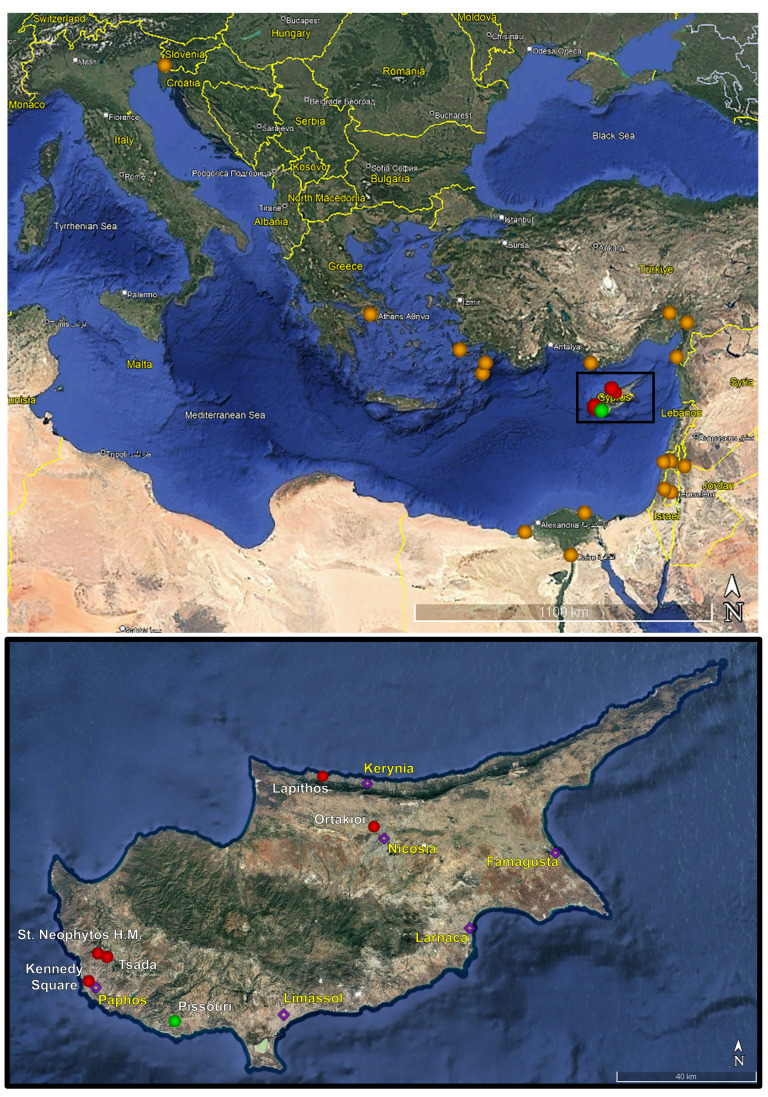
**Above**: Map of the eastern Mediterranean showing the known distribution of *S.ioanniticus*, indicated by **orange spheres**. The **black rectangle** highlights Cyprus's location. **Below**: Map of Cyprus; **Green sphere with white text**: Confirmed record from Pissouri Village; **Red spheres with white text**: Unconfirmed Amblypygi records from literature and citizen-science sources; **Violet diamonds with yellow text**: Major cities of Cyprus.

A map with the above records and the known distribution of *S.ioanniticus*, based on the available literature, is shown in Fig. 3 (El-Hennawy 2002,Agapakis and de Miranda 2019, Shakhatreh et al. 2020, Miranda et al. 2021).

## Discussion

This article officially confirms the presence of Amblypygi on the island of Cyprus, after eight years of the first sighting of specimens. This first record of *S.ioanniticus* in Cyprus adds one more order of organisms (Amblypygi), a new family (Charontidae), a new genus (*Sarax*) and a new species to the island’s fauna, thus filling gaps in our knowledge of the arachnids of Cyprus and supplementing data on their distribution and ecology. The presence of an Amblypygi on Cyprus is an important addition to its already rich arachnid fauna, including a diversity of endemic and interesting species (Gantenbein et al. 2000, Yagmur 2011, Bosmans et al. 2016, Azarkina et al. 2018, Bosmans et al. 2019, Bayoumy et al. 2024).

Up-to-date reports of Amblypygi in Cyprus have been obtained from the south-western and north-western parts of the island, at a maximum distance of 90 kilometres apart. This fact, in combination with Sket (1990), leads us to assume that *S.ioanniticus* holds established populations in Cyprus. The presence of the species in Cyprus was expected, based on the known distribution on the surrounding mainland that spreads from Italy to Egypt (Miranda et al. 2021). Furthermore, its confirmed presence on the island shows that the species is more widely distributed than expected, but is rarely observed due to its “cryptic” behaviour and ecological requirements. Taking into account the synanthropic nature of *S.ioanniticus* (Blick and Harvey 2011, Miranda et al. 2016, Agapakis and de Miranda 2019, current study), its parthenogenetic reproduction ability (Weygoldt 2007, Blick and Harvey 2011, Blick and Seiter 2016, Miranda et al. 2016) and the recent increase in citizen-science engagement (Goodchild 2007, Dickinson et al. 2012), more observations are anticipated across the Mediterranean Region.

Citizen science has played a crucial role in documenting species such as *S.ioanniticus* in Cyprus and the broader Mediterranean Region. Local contributions, especially through platforms like iNaturalist and social media groups focused on biodiversity, have been instrumental in recording occurrences of numerous important alien and native species. Such records, contributed by non-professionals, help fill gaps in species’ distributions, offer valuable support for ecological research and assist conservation efforts. In Cyprus, citizen-science data have contributed to significant findings, underscoring the importance of public engagement in preserving biodiversity (Maistrello et al. 2016, Chandler et al. 2017, Demetriou et al. 2020, Johnson et al. 2020, Kazilas et al. 2020, Ruzzier et al. 2020, Davranoglou and Karaouzas 2021, Hadjiconstantis and Zoumides 2021, Kazilas et al. 2021, Angelidou et al. 2022, Demetriou et al. 2022, Christou et al. 2023, Hadjiconstantis et al. 2023, John et al. 2023, González‐Moreno et al. 2024).

In conclusion, more research on the distribution, ecological preferences, species interrelationships and threats *S.ioanniticus* may face in Cyprus are necessary to monitor and safeguard this peculiar, rarely encountered species.

## Supplementary Material

XML Treatment for Sarax ioanniticus
